# Over-expression of protein tyrosine phosphatase 4A2 correlates with tumor progression and poor prognosis in nasopharyngeal carcinoma

**DOI:** 10.18632/oncotarget.20550

**Published:** 2017-08-24

**Authors:** Ying Gao, Mengping Zhang, Zhousan Zheng, Ying He, Yujia Zhu, Quanyong Cheng, Jian Rong, Huiwen Weng, Cui Chen, Yi Xu, Miao Yun, Jiaxing Zhang, Sheng Ye

**Affiliations:** ^1^ Department of Oncology, The First Affiliated Hospital, Sun Yat-sen University, Guangzhou, PR China; ^2^ Sun Yat-sen University Cancer Center, State Key Laboratory of Oncology in South China, Collaborative Innovation Center for Cancer Medicine, Guangzhou, PR China; ^3^ Departments of Radiation Oncology, State Key Laboratory of Oncology in South China, Collaborative Innovation Center for Cancer Medicine, Sun Yat-sen University Cancer Center, Guangzhou, Guangdong, PR China; ^4^ Department of Private Surgery Medical Center, The First Affiliated Hospital, Sun Yat-sen University, Guangzhou, PR China; ^5^ Department of Extracorporeal Circulation, The First Affiliated Hospital, Sun Yat-sen University, Guangzhou, PR China; ^6^ Department of Ultrasound, State Key Laboratory of Oncology in South China, Collaborative Innovation Center for Cancer Medicine, Sun Yat-sen University Cancer Center, Guangzhou, PR China

**Keywords:** protein tyrosine phosphatase 4A2(PTP4A2), nasopharyngeal carcinoma, tumor progression, prognosis, biomarker

## Abstract

Protein tyrosine phosphatase 4A2 (PTP4A2) has been implicated as an oncogenic protein in several human cancers. However, the level of PTP4A2 expression and its prognostic significance in nasopharyngeal carcinoma (NPC) remains unknown. In this study, Western blotting (WB), quantitative real-time PCR (qT-PCR) and immunohischemistry (IHC) was applied to evaluated the expression levels of PTP4A2 in NPC cell lines and tumor tissues combining two independent cohorts. Receiver-operator curve (ROC) analysis was used to assessed the optimal cut-off score in training cohort (266 cases). This cut-off score was subjected to determine the association of PTP4A2 expression with patients’ clinical characteristics and survival outcome in the validation cohort (201 cases) and the overall population (467 cases). We found that PTP4A2 were significantly overexpressed in NPC cell lines compared with normal nasopharyngeal epithelial cell. Moreover, overexpression of PTP4A2 was positively correlated with advanced T classification (*P<0.001*) and TNM stages (*P<0.001*). And higher PTP4A2 expression was an independent prognostic factor for adverse overall survival (*P<0.05*) and poor disease-free survival (*P<0.05*). Our results demonstrated that the overexpression of PTP4A2 was closely associated with poor survival outcome in patients with NPC and may represent a novel prognostic biomarker and therapeutic target for this disease.

## INTRODUCTION

Nasopharyngeal carcinoma (NPC), an Epstein-Bar virus (EBV)-related head and neck cancer, is a neoplastic disorder that arises from the epithelial lining of the nasopharynx [1]. Though rarely seen in western countries, it is commonly found in endemic areas such as South East Asia and Southern China [2]. Although radiotherapy with concomitant chemotherapy has increased the survival outcome of early-stage NPC, local recurrences and distant metastasis are still the vital issues for the poor outcome of patients at advanced-stage [3]. Despite improvements in the management of patients with NPC over the years, it still poses a significant medical challenge with 84 thousand newly diagnosed cases with 51 thousand deaths globally in 2008 [4]. The conventional Tumor Node Metastasis (TNM) stage system is inadequate in precisely predicting patients’ survival, thus there is still an urgent need to identify promising diagnostic and new therapeutic targets to help in the management of NPC.

Tyrosine phosphorylation and dephosphorylation, are ubiquitous regulatory events affecting the functional activities of protein that activate diverse cellular processes, such as cell cycle progression, apoptosis, transcriptional regulation, protein trafficking and protein degradation [5]. Altered activity of protein tyrosine phosphatases (PTPs) were revealed as a result of aberrant protein tyrosine phosphorylation. Accumulating evidence supported that enhanced expression of members of the human phosphatase of regenerating liver(PRL) subgroup of PTPs is linked to the progression of human cancer. This family are encoded by the *PTP4A1*, *PTP4A2*, and *PTP4P3* genes, respectively [6, 7]. Previous studies have showed that up-regulation of the PRLs, especially PTP4A1 and PTP4A3 could promote cell invasion, migration and metastasis during tumor development and progression [8–10]. However, few studies have reported the specific functions of PTP4A2 in human cancer types. Recently, the expression level of PTP4A2 was found to be significantly upregulated in several types of malignancies, especially in breast, colon and pancreatic cancer and could be a potential biomarker for these diseases [11–13]. Of note, we reported previously PTP4A2 was up-regulated in colorectal cancer (CRC) and associated with CRC metastasis [11]. However, to the best of our knowledge, the expression pattern of PTP4A2 and its clinical significance in NPC remains inconclusive.

In this current study, we examined the expression status of PTP4A2 in two independent cohorts of NPC patients using immunohistochemistry (IHC) method. Receiver operating characteristic (ROC) curve analysis was performed to select the optimal cut-off value for PTP4A2 expression into high- and low-expression groups. We finally founded that up-regulated expression of PTP4A2 was significantly associated with aggressive clinical features and poor survival of NPC patients in both the training and validation cohorts.

## RESULTS

### Up-regulation of PTP4A2 in NPC cell lines and tissues

**Figure 1:**
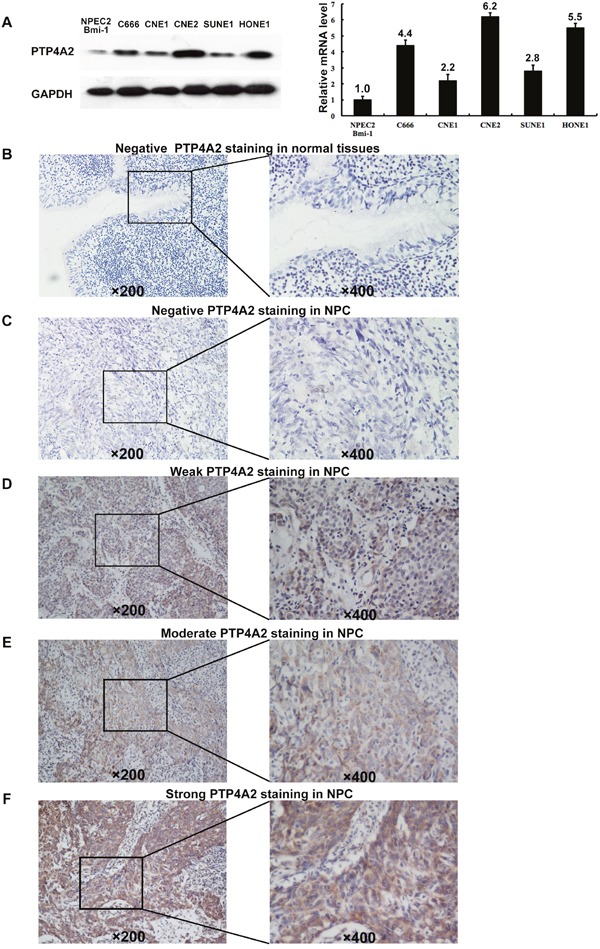
Western blotting, qPCR and IHC assay of the expression of PTP4A2 in NPC cell lines and tissues (**A**) Western blot (left panel) and Quantitative RT-PCR (right panel) assay of PTP4A2 protein expression in NPEC2 Bmi-1 and 5 NPC cell lines. GADPH was used as a loading control. (**B**) Normal nasopharyngeal mucosa tissue showed negative expression of PTP4A2 protein (upper panel×200). (**C**) Representative image of negative (Scoring intensity =0) (**C**), weak (Scoring intensity =1) (**D**), moderate (Scoring intensity=2) (**E**) and strong (Scoring intensity=3) (**F**) PTP4A2 IHC staining in NPC tissues is shown (upper panel×200). The lower panels indicated the higher magnification (×400) from the area of the box in B, C, D, E and F, respectively. Data in A-F were obtained from independent triplicate experiments with similar results. *p < 0.05.

Western blotting and quantitative real-time PCR was applied to analysis the expression levels of PTP4A2 protein and mRNA in five NPC cell lines (CNE1, CNE2, C666, HONE1 and SUNE1) and one immortalized primary nasopharyngeal epithelial cell line (NPEC2 Bmi-1). Our western blotting analysis demonstrated that NPC cell lines exhibited higher level of PTP4A2 protein expression as compared to that in NPEC2 Bmi-1 (Figure 1A: left). qRT-PCR also revealed elevated expression of PTP4A2 mRNA in CNE1, CNE2, C666, HONE1 and SUNE1 compared to NPEC2 Bmi-1 (Figure 1A: right). In addition, IHC analysis was then conducted to determine PTP4A2 expression level in two independent cohorts of NPC tissue specimens. For PTP4A2 IHC staining, the positive immunoreactivity was observed primarily in the cytoplasm of NPC cells, and the representative four categories of the intensity of PTP4A2 immunostaining were observed in Figures 1B-1F. Consistently, IHC staining showed that PTP4A2 is up-regulated in tumor tissues compared with the corresponding adjacent non-neoplastic nasopharyngeal tissues (ANTs). These results indicate that PTP4A2 was overexpressed in NPC.

### Selection of the cutoff score for PTP4A2 expression

**Figure 2:**
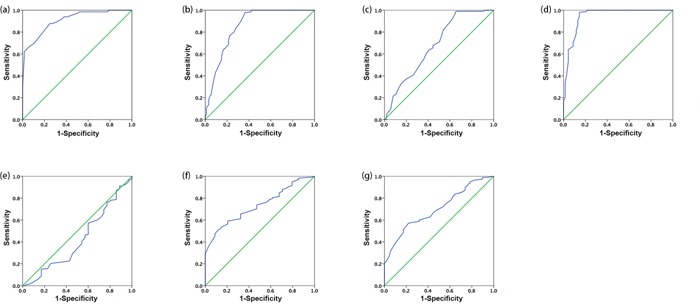
Receiver-operator curves(ROC) were used to determine the cut-off scores for positive expression of PTP4A2 The sensitivity and specificity for each outcome were plotted: survival status **(a)**, pT status **(b)**, pN status **(c)**, pTNM stage **(d)**, WHO type **(e)**, VCA-IgA **(f)**, and EA-IgA **(g)** in training cohort.

To better estimate the expression of PTP4A2 in NPC, we first subjected the IHC score of PTP4A2 in the training cohort to ROC curve analysis with respect to each clinical feature (Figure 2). Cancers with scores above the obtained cut-off value were considered to have high PTP4A2 expression, which led to the greatest number of cancers correctly classified, based on having or not having the clinical outcome. The corresponding AUCs (95% confidence interval [CI]) are listed in Table 1. As shown in Figure 2. and Table 1, the AUC for survival status had the biggest area. So we selected 180 as the optimal cut-point for survival analysis. Base on this cut point, high expression of PTP4A2 could be detected in 108/226 (40.6%) of training cohort and 99 of 201 (49.3%) of validation cohort, respectively.

**Table 1:** Area under the receiver-operator curve for each clinicopathological feature

Feature	AUC (95% CI)	*P*-value
**Survival status**	0.907(0.866-0.948)	<0.001
**T status**	0.853(0.799-0.906)	<0.001
**N status**	0.679(0.616-0.743)	<0.001
**TNM stage**	0.945(0.907-0.982)	<0.001
**WHO type**	0.423(0.316-0.531)	0.144
**VCA-IgA**	0.732(0.658-0.806)	<0.001
**EA-IgA**	0.703(0.637-0.768)	<0.001

AUC, area under the curve; CI, confidence interval.

### The relationship between PTP4A2 expression and the clinicopathologic features of NPC patients in two independent cohorts

Table 2. showed the detailed information of NPC patients’ clinicopathologic features and the correlation between the high PTP4A2 expression and the two cohorts of patients. In training cohort, we observed that a significant correlation between PTP4A2 expression with T classification (*P*<0.001), N status(*P*=0.003), TNM stage(*P*<0.001), Viral capsid antigen immunoglobulin A (VCA IgA) (*P*<0.001) and Early antigen immunoglobulin A (EA IgA) (*P*<0.001). But in validation cohort, the difference was only observed in T classification (*P*=0.002) and TNM stage (*P*=0.012). As expected, we failed to discover a significant difference between PTP4A2 expression and other clinical characteristics in both cohorts, including patients’ age, gender and WHO type (*P*>0.05).

**Table 2:** The association of PTP4A2 expression with clinicopathological variables in both cohorts

Variable	Testing Cohort				Validation Cohort			
	Case	Low expression	High expression	*P* value^a^	Case	Low expression	High expression	*P* value^b^
**Age**				0.477				0.963
**≤45^a^**	140	86	54		89	44	45	
**>45**	126	72	54		112	55	57	
**Gender**				0.157				0.069
**Female**	61	41	20		63	37	26	
**Male**	205	117	88		138	62	76	
**T classification**				<0.001*				0.002*
**T1-2**	94	80	14		74	47	27	
**T3-4**	172	78	94		127	52	75	
**N status**				0.003*				0.855
**N0-1**	173	114	59		115	56	59	
**N2-3**	93	44	49		86	43	43	
**TNM stage**				<0.001*				0.012*
**I-II**	69	66	3		35	24	11	
**III-IV**	197	92	105		166	75	91	
**WHO type**				0.077				0.730
**Type I+ II**	35	16	19		7	3	4	
**Type III**	231	142	89		194	96	98	
**VCA IgA**				<0.001*				0.874
**<1:80**	34	31	3		21	10	11	
**≥1:80**	232	127	105		180	89	91	
**EA IgA**				<0.001*				0.216
**<1:10**	69	57	12		55	31	24	
**≥1:10**	197	101	96		146	68	78	

a: Mean age

b: Chi-square test

*Statistically significant difference

### The relationship between clinicopathologic variables, PTP4A2 expression and NPC patient survival: Univariate survival analysis

**Figure 3:**
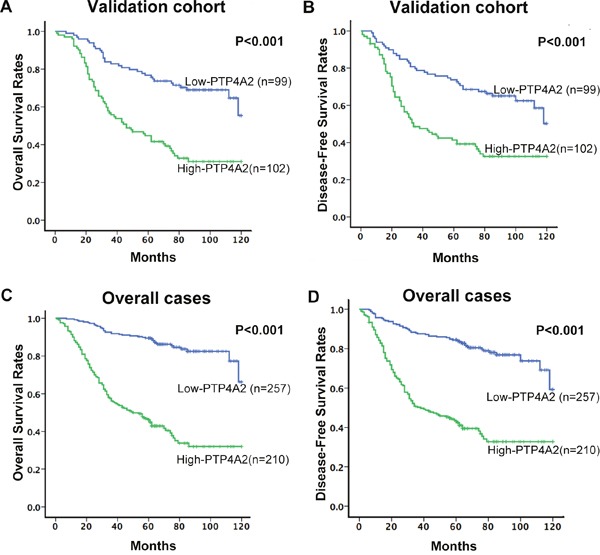
The association between PTP4A2 expression and NPC patients’ survival Kaplan-Meier survival analysis of PTP4A2 expression for Overall Survival and Disease-Free Survival in validation cohort **(A** and **B)** and in overall cases **(C** and **D)** (long-rank test).

The established prognostic factors of the NPC patients’ survival were first utilized to test the representativeness of all NPC cases in our study. Univariate analysis suggested a significant impact of well-known clinicopathologic prognostic features, such as such as T classification, N status and TNM stage on the patient survival rates (*P*<0.05, Table 3). Survival assessment also revealed that increased expression of PTP4A2 correlated with was adverse overall survival (OS) (*P<0.001*, Figure 3; hazard ration (HR)=5.957, 95%CI: 4.157-8.853, *P<0.001*, Table 3). Disease free survival (DFS) analysis result was similar to OS analysis. Patients with increased PTP4A2 expression also showed a significant trend toward worse DFS compared to those with normal PTP4A2 expression (*P<0.001*, Figure 3; HR=4.349, 95%CI: 3.158-5,988, *P<0.001*, Table 4). In the validation cohort, similar results were observed. With regarding to OS, patients with high PTP4A2 expression also has a significant worse trend compared to those with low expression (*P<0.001*, Figure 3; HR=2.973, 95%CI: 1.944-4.545, *P<0.001*, Table 3). For DFS, a similar trend could also be observed (*P<0.001*, Figure 3; HR=2.512, 95%CI: 1.674-3.771, *P<0.001*, Table 4.). Of the other prognostic factors, univariate analysis for validation cohort confirmed that the traditional TNM stage was useful predictor for patients’ OS (*P<0.05*, Table 3) and DFS (*P<0.05*, Table 4)

**Table 3:** Univariate analysis of PTP4A2 expression and various clinicopathological parameters in validation and overall cases of NPC patients for Overall Survival

Variables	Validation cohort				Overall cases			
	Case	HR	95%CI	*P*-value	Case	HR	95%CI	*P*-value
**Age**				0.282				0.061
**≤45**	89	1.0			229	1.0		
**>45**	112	1.248	0.833-1.868		238	1.343	0.986-1.829	
**Gender**				0.074				0.661
**Female**	63	1.0			124	1.0		
**Male**	138	1.512	0.961-2.380		343	1.081	0.763-1.531	
**T status**				0.077				<0.001*
**T1-2**	74	1.0			168	1.0		
**T3-4**	127	1.465	0.960-2.237		299	2.006	1.411-2.852	
**N status**				0.206				**0.003**
**N0-1**	115	1.0			288	1.0		
**N2-3**	86	1.290	0.869-1.914		179	1.589	1.170-2.158	
**TNM stage**				0.008*				<0.001*
**I-II**	35	1.0			104	1.0		
**III-IV**	166	2.432	1.264-4.681		363	3.914	2.262-6.773	
**WHO type**				0.453				0.116
**Type I+ II**	7	1.0			42	1.0		
**Type III**	194	0.681	0.250-1854		425	0.682	0.442-1.100	
**VCA IgA**				0.244				0.718
**<1:80**	21	1.0			55	1.0		
**≥1:80**	180	0.707	0.394-1.267		412	0.919	0.581-1.453	
**EA IgA**				0.376				0.924
**<1:10**	55	1.0			124	1.0		
**≥1:10**	146	0.822	0.533-1.268		343	1.017	0.720-1.436	
**PTP4A2 expression**				<0.001*				<0.001*
**Low expression**	99	1.0			257	1.0		
**High expression**	102	2.973	1.944-4.545		210	5.957	4.157-8.853	

Cox proportional hazard regression model, enter; HR, Hazard ratio; CI, confidence interval;

*Statistically significant difference

**Table 4:** Results of Univariate Cox proportional-hazards analysis of PTP4A2 expression and various clinicopathological parameters in validation and overall cases of NPC patients for Disease-Free Survival

Variables	Validation cohort				Overall cases			
	Case	HR	95%CI	*P*-value	Case	HR	95%CI	*P*-value
**Age**				0.284				0.112
**≤45**	89	1.0			229	1.0		
**>45**	112	0.806	0.543-1.196		238	1.267	0.946-1.697	
**Gender**				0.101				0.513
**Female**	63	1.0			124	1.0		
**Male**	138	1.444	0.931-2.241		343	1.117	0.802-1.556	
**T status**				0.722				<0.001*
**T1-2**	74	1.0			168	1.0		
**T3-4**	127	0.722	0.479-1.088		299	1.963	1.410-2.733	
**N status**				0.181				<0.001*
**N0-1**	115	1.0			288	1.0		
**N2-3**	86	1.302	0.884-1.918		179	1.700	1.272-2.273	
**TNM stage**				0.019*				<0.001*
**I-II**	35	1.0			104	1.0		
**III-IV**	166	2.060	1.127-3.766		363	3.565	2.162-5.876	
**WHO type**				0.484				0.177
**Type I+ II**	7	1.0			42	1.0		
**Type III**	194	0.700	0.257-1.903		425	0.726	0.456-1.156	
**VCA IgA**				0.177				0.934
**<1:80**	21	1.0			55	1.0		
**≥1:80**	180	0.669	0.374-1.198		412	1.020	0.647-1.607	
**EA IgA**				0.389				0.782
**<1:10**	55	1.0			124	1.0		
**≥1:10**	146	0.829	0.542-1.269		343	1.048	0.753-1.456	
**PTP4A2 expression**				<0.001*				<0.001*
**Low expression**	99	1.0			257	1.0		
**High expression**	102	2.512	1.674-3.771		210	4.349	3.158-5.988	

Cox proportional hazard regression model, enter; HR, Hazard ratio; CI, confidence interval;

*Statistically significant difference

### Independent prognostic factors of NPC: Multivariate Cox regression analysis

The PTP4A2 expression as well as other clinicopathologic features that displayed significant impact on patient survival based on univariate analyses was examined in multivariate analysis. We found that high expression of PTP4A2 was an independent prognostic factor for poor OS of NPC patients in both validation cohort and overall cases (HR=2.784,95%CI: 1.815-4.269, *P<0.001*; HR=5.065, 95%CI: 3.490-7.351, *P<0.001*, respectively, Table 5) as evidenced by multivariate analysis. The same results were obtained when considered patients’ DFS (HR=2.385, 95%CI: 1.585-3.589, *P<0.001*; HR=3.669, 95%CI:2.635-5.110, *P<0.001*, respectively, Table 6). Of other features, TNM stage was found to be an independent prognostic predictor for poor OS (*P*<*0.05*, Table 5) in both validation cohort and overall cases. But for DFS, the TNM stage has no significant value as an independent factor in validation cohort (HR=1.790, 95%CI= 0.976-3.286, *P=0.060*, Table 6).

**Table 5:** Results of multivariate Cox proportional-hazards analysis for Overall Survival for NPC patients

Variable	HR	95%CI	*P*-value
**Validation cohort**			
TNM stage(I+II vs. III+IV)	2.033	1.051-3.931	0.035*
PTP4A2 expression(Low vs. High)	2.784	1.815-4.269	<0.001*
**Overall cases**			
TNM stage(I+II vs. III+IV)	2.106	1.192-3.720	0.010*
PTP4A2 expression(Low vs. High)	5.065	3.490-7.351	<0.001*

Cox proportional hazard regression model, enter; HR, Hazard ratio; CI, confidence interval;

*Statistically significant difference

**Table 6:** Results of multivariate Cox proportional-hazards analysis for Disease-Free survival for NPC patients

Variable	HR	95%CI	*P*-value
**Validation cohort**			
TNM stage(I+II vs. III+IV)	1.790	0.976-3.286	0.060
PTP4A2 expression(Low vs. High)	2.385	1.585-3.589	<0.001*
**Overall cases**			
TNM stage(I+II vs. III+IV)	2.184	1.300-3.667	0.003*
PTP4A2 expression(Low vs. High)	3.669	2.635-5.110	<0.001*

Cox proportional hazard regression model, enter; HR, Hazard ratio; CI, confidence interval;

*Statistically significant difference

## DISCUSSION

NPC is a common malignancy and a major public health problem in endemic regions. At present, TNM stage is accepted as prognostical indicator for NPC. However, patients with the same TNM stage of NPC sometimes show considerable variability in tumor recurrence and metastasis. Thus, it will be meaningful to search for novel biomarkers that can provide additional staging information to assist the choice of therapy and optimize treatment outcomes. Previous studies have revealed several abnormally expressed genes in NPC that could serve as reliable prognostic factors, like H3K27me3, ULK1, LOX, Talin-1 and PTP4A3 [8, 14–17], however, the existing molecular markers are substantially limited in identifying tumor spread and aiding risk assessment.

Protein tyrosine phosphates (PTP) constitutes a large family of enzymes that can exert variable effects on signaling pathways [18, 19]. Noteworthy, as observed with protein kinases, perturbations in tyrosine phosphorylation underlie many human diseases, particularly in cancer [20]. PTP4A1, PTP4A2, and PTP4P3 are three closely related small PTPs which drawn attention to us about their functions in tumor cell proliferation, including promotion of tumor migration, invasion, and metastasis [8–10, 21]. Recently, accumulating studies showed that up-regulated expression of PTP4A2 correlated significantly with cancer progression in several types of malignancies [11–13]. We have not seen the status of PTP4A2 expression and its correlation with some clinicopathological parameters been investigated in NPC though. In the present study, higher PTP4A2 protein and mRNA levels in NPC cells were seen by western blotting and qRT-PCR have shown when compared to that in immortalized human nasopharyngeal epithelial cells. An up-regulation in NPC tissues was also seen by immunohistochemistry compared with that in ANTs. In two large cohorts of nasopharyngeal carcinoma samples, overexpression of PTP4A2 protein was observed in 40.6% (108/266) and 50.7%(102/201) of NPC tissues, respectively. These results suggest that overexpression of PTP4A2 may provide a selective advantage in the NPC tumorigenic processes. Then we observed that high expression of PTP4A2 was significantly associated with T classification and TNM staging, which were often recognized as aggressive clinical features. These finding were consistent with other studies. For example, Zhao *et al* and Andres *et al* separately reported the high expression of PTP4A2 protein in human breast cancer, where was positively correlated with tumor aggressiveness and/or advanced clinical stage [12, 22]. Our current finding supported that the expression of PTP4A2 increases with NPC progression and this gene may be acted as an oncogene in the development and progression of NPC. Collectively, these data suggest that PTP4A2 expression may function as on oncogene of malignant transformation in human cancers. The most important finding of our study is the prognostic significance of PTP4A2 expression in NPC. The Kaplan-Meier analysis and log rank test demonstrated that up-regulated PTP4A2 expression was significantly associated with adverse OS and poor DFS. More importantly, we further demonstrated that PTP4A2 is an independent prognostic factor for OS and DFS in NPC. Similar findings have been reported in the prognostic impact of PTP4A2 on other human cancers, such as human breast cancer [12] and colony cancer [11]. In particular, high PTP4A2 expression was found to be related with shorter OS and DFS in breast cancer patients [12, 19, 22]. Our present findings suggested that IHC method, detected as an examination of PTP4A2 expression, could be a reliable tool to predict the prognostic outcome of NPC and make the optimal clinical decisions. For example, those high-risk patients with high PTP4A2 expression might be considered for higher-dose radiation or chemotherapy. By contrast, low-risk NPC patients with lower PTP4A2 expression, might benefit from milder treatment options and avoid excessive radical therapies. However, further investigation is required before a clinical practice may be recommended.

As we known, the majority of cancer deaths is a result of tumor metastasis rather than primary tumors, and the vascular invasion and metastasis are important steps in cancer progression. The potential function of PTP4A2 in cancer progression have been explored in recently investigations. A study by James *et al* demonstrated that PTP4A2 might activate small GTPases of the Rho family to promote invasion and metastasis [7]. Other study showed that PTP4A2 overexpression promotes breast cancer progression likely via the ERK1/2 pathway, and PTP4A2 may acted as a regulator of RabGGT II activity, this phosphatase might also have a relationship with Rab-regulated recycling pathway that contribute to cell transformation and migration [19]. Wang *et al* showed that PTP4A2 could regulated tumor cell migration and invasion in human lung cancer cells through an ERK-dependent signaling pathway [23]. We previously discovered that PTP4A2 could activate AKT/GSK3β/β-catenin pathways to induce epithelial-to-mesenchymal transition (EMT) during colorectal cancer metastasis [24]. All these study illustrated that the function of PTP4A2 in human cancer might be tissue-specific. Although in present study, we identified the correlation of PTP4A2 expression in cancer and NPC patients’ survival, more works are needed in the future study to elucidate the mechanisms by which PTP4A2 is involved in the development and progression of NPC.

In summary, this is the first study aimed at evaluating the possible use of PTP4A2 as a clinically relevant indicator for NPC aggressiveness and as a prognostic marker for patient survival in NPC. Nevertheless, further investigation into the cellular pathways by which PTP4A2 exerts its influence on the development and progression of NPC and prospective studies on the prognostic significance of PTP4A2 are required. We hope PTP4A2 might be helpful to render a more personalised treatment strategy in NPC.

## MATERIALS AND METHODS

### Cell lines and cell cultures

NPC cell lines (CNE1, CNE2, C666, HONE1 and SUNE1), and one immortalized human nasopharyngeal epithelial cells (NPECs) induced by Bmi-1 (NPEC2 Bmi-1) were obtained from the group of Musheng Mu. The cells were cultured according to the instructions from the supplier. They were maintained in RPMI-1640 medium supplemented with 10% fetal bovine serum (FBS) and 1% penicillin-streptomycin at 37°C with 5% CO_2_.

### Patients and Follow-up

Paraffin-embedded pathological specimens of NPC patients(n=266) in training cohort were obtained form the archives of the Department of Pathology, between January 2002 and December 2004 at Sun Yat-sen University Cancer Center, Guangzhou, China. Another randomly collected, independent validation cohort of 201 NPC patients between January 1998 and December 2001 from the same department were obtained and assessed in parallel. The enrollment criteria were: 1) histopathologically and clinically proven primary carcinoma of nasopharynx; 2) no previous malignant disease or a second primary cancer; 3) no previous treatment or severe complications. The clinicopathologic information of two cohorts including age; gender; T classification; N status; TNM stage; WHO type; Viral capsid antigen immunoglobulin A (VCA IgA) and Early antigen immunoglobulin A (EA IgA) were described in Table 2. The follow-up data were obtained by telephone or from the outpatient records. Overall survival (OS) was defined as the time (in months) from the date of admission to the date of NPC-related death. Disease-Free Survival (DFS) was defined as the interval between the date of admission and tumor recurrence (including local-regional relapse or distant metastasis), if disease recurrence was not diagnosed, patients were censored at the date of death or last follow-up. For surviving patients, the data were censored at the last follow-up. Deaths from other causes were treated as censored cases. Clinical stage of each case was done according to the TNM classification system established by the American Joint Committee on Cancer/International Union Against Cancer. Follow-up assessments were performed every 3 months for the first 2 years, followed by every 6 months until the patient succumbed. The tumor recurrence was confirmed by magnetic resonance imaging(MRI) and/or contrast computed tomography(CT). The study protocol was approved by the Ethics Committee of Sun Yat-sen University, Guangzhou, China. Informed consent was obtained from each patient. All experimental methods were carried out in accordance with approved guidelines of Sun Yat-sen University Cancer Center.

### Immunohistochemistry(IHC) analysis

The expression of PTP4A2 in NPC was examined by immunohistochemical techniques using a standard two-step technique as demonstrated previously [25]. These slides were deparaffinized in xylene, rehydrated through a graded alcohol series (100%,95%, and 75% in sequence). The endogenous peroxidase activity was block by immering samples in 3% hydrogen peroxide for 20 minutes and antigen retrieval was performed by microwave heating for 15 minutes in 10 nM citrate buffer (PH=6.0). Then, the slides were incubated with 10% normal goat serum at room temperature for 10 minutes to reduce nonspecific reactivity. Subsequently, the slides were incubated with rabbit polyclonal antibody against PTP4A2 (Abcam, Cambridge, MA,1: 200 dilution) and stored overnight at 4°C. After washing three times with 0.01mol/L phosphate-buffered saline (PBS; PH=7.4) for 5 minutes, the slides were incubated with a secondary antibody (Envision; Dako, Glostrup, Demark) for 1 hour at room temperature. Thereafter, the sections were stained with 3,3-diaminobenzidine (DAB). Finally, the sections were counterstained with Mayer's hematoxylin, dehydrated, and mounted. Phosphate-buffered saline (PBS) was used to replaced anti-PTP4A2 antibody as a negative control.

### IHC evaluation

Expression levels of PTP4A2 protein were evaluated by microscopic examination of stained slides. The presence of cytoplasmic brown granules was considered to be positive for PTP4A2 expression. In Brief, the expression pattern was assessed as follows: the staining intensity was assigned as 0 (no staining), 1+ (weak), 2+ (moderate) or 3+ (strong) (I0-3). Then, the proportion of tumor cells with that intensity was divided by the total number of tumor cells and recorded in 5% increments from 0 to 100 (P0, P1-3). Each IHC score (range 0-300) was determined by adding the sum of the scores obtained for each intensity and the proportion of the area stained (F=I1×P1+I2×P2+I3×P3). The reproducibility of the scoring manner has been described previously [26]. PTP4A2 expression was assessed by three independent pathologists who were blinded to the clinicopathological data. The conclusions of the pathologists were in complete agreement in approximately 85% of the cases, which confirmed that this scoring method was highly reproducible. Three pathologists worked together to confirm the scores when two out of three independent results appeared to be different.

### Selection of cutoff score

Receiver-operator curve (ROC) analysis was applied to PTP4A2 to define cutoff scores for tumor “positivity” by a 0,1-criterion [27]. Briefly, the sensitivity and specificity for the evaluated outcome were plotted to create various ROC curves. The score closest to the point with the maximum sensitivity and specificity (i.e.,0.0,1.0) was selected as the cutoff score to determine the greatest number of tumors that were correctly classified as having or not having the outcome [28, 29]. To facilitate the ROC curve analysis, the clinicopathologic characteristics were dichotomized as follow: T classification (T1-2 vs. T3-4), N status (N0-1 vs. N2-3), TNM stage (I+II vs. III+IV), WHO type (I+II vs. III), VCA-IgA (<1:80 vs. ≥1:80), EA-IgA (<1:10 vs. ≥1:10) and survival status [death due to NPC vs. censored (lost to follow up, alive or death from other causes)].

### Western blotting analysis

Whole cell extracts were prepared in radio immunoprecipitation assay (RIPA) buffer and centrifuged at 12000g for 15 minutes. Protein concentrations were measured using the bicinchoninic acid assay. Equal amounts of whole cell lysates were separated by SDS-polyacrylamide gel electrophoresis (PAGE) and electrotransferrend on a ployvinylidene difluoride (PVDF) membrane (Pall Corp., Port Washington, NY). The membranes were then blocked with 5% skimmed milk and incubated with primary rabbit monoclonal antibodies against PTP4A2 (Abcam, Cambridge, MA, USA, 1: 1000 dilution) and GAPDH (Abcam, Cambridge, MA, USA, 1:1000 dilution), respectively. After washing the membrane, it was then incubated with secondary anti-mouse antibody from Santa Cruz Biotechnology (Santa Cruz Biotechnology, CA, USA). The immunoreactive signals were detected with enhanced chemiluminescence kit (Amersham Biosciences, Uppsala, Sweden). The procedures followed were conducted in accordance with the manufacturer's instructions.

### Quantitative real-time polymerase chain reaction (qRT-PCR)

Total RNA was extracted from the NPC cell lines using Trizol regent (Invitrogen, Grand Island, NY, USA) and cDNA was synthesized by SuperScript Reverse Transcriptase kit (Promega, Madison, WI, USA) according to the manufacturer's instruction. The primer sequences used to amplify PTP4A2 were: 5′-AGCCAGGTTGCTGTGTTGCAG-3′ (forward) and 5′-CACAGCAATGCCCATTGGTA-3′ (reverse). GAPDH was used as an internal control for normalization.

### Statistical analysis

Statistical analysis was performed with the SPSS statistical software package (standard version 19.0; SPSS, Chicago, IL). The relationship between PTP4A2 expression and the clinicopathologic features of the NPC patients in both cohorts were evaluated by a Pearson's chi-squared test. ROC analysis was employed to determine the cutoff value for PTP4A2 positivity. Univariate and multivariate survival analysis was performed with the Cox proportional hazards regression model. The corresponding Hazard ratio (HR) and 95% CI were taken from Cox regression models. Survival curves were plotted by the Kaplan-Meier method and compared by the log-rank test. Differences were considered significant if the P-value from a two-tailed test was<0.05.

## Abbreviations

PTP4A2: protein tyrosine phosphatase 4A2
NPC: nasopharyngeal carcinoma
NPECs: nasopharyngeal epithelial cells
IHC: immunohistochemistry
ROC curve: Receiver operating characteristic curve
OS: overall survival
DFS: disease-free survival
VCA-IgA: viral capsid antigen immunoglobulin A
EA-IgA: early antigen immunoglobulin A
TNM: Tumor-lymph node-metastasis
WHO: World Health Organization
ANT: adjacent normal tissue
